# Effect and mechanism of modified Yougui power on Simmental bulls with oligoasthenozoospermia based on targeted amino acid metabolism

**DOI:** 10.3389/fvets.2025.1595145

**Published:** 2025-06-18

**Authors:** Baoxia Chen, Qiang Ma, Huifang Ma, Wenfei Zhang, Runmin Wu, Chun Niu, Rongxia Guo, Zhiyuan Ma, Peng Ji, Yanming Wei, Yongli Hua

**Affiliations:** ^1^College of Veterinary Medicine, Institute of Traditional Chinese Veterinary Medicine, Gansu Agricultural University, Lanzhou, China; ^2^Gansu Provincial Livestock Breeding and Improvement Management Station, Wuwei, China

**Keywords:** modified Yougui Power, Simmental bulls, oligoasthenozoospermia, amino acid metabolism, molecular docking

## Abstract

**Objective:**

Oligoasthenozoospermia (OA) is a common reproductive disorder characterized by reduced sperm count and motility in animals. Yougui Pill (YP) is a traditional Chinese medicine formula for the treatment of oligoasthenozoospermia. However, its effects on Simmental bulls are relatively limited, and the mechanisms involved in the regulation of OA remain unknown.

**Methods:**

In this study, antler gum was removed from the original formula, and the key components and their mechanism of action of Modified Yougui Power (MYP) for the treatment of OA were investigated by UPLC-MS/MS analysis, amino acid metabolomics studies, and molecular docking analysis. UPLC-MS/MS was used to detect and study the active compounds of MYP. The levels of T, E2, FSH, and LH in the serum of OA and the control group were detected by enzyme-linked immunosorbent assay (ELISA). The levels of amino acid metabolites and related metabolic pathways in semen of the OA and control groups were detected by UHPLC-MRM-MS/MS. Molecular docking was used to assess the affinity between the primary active ingredients associated with OA and their core targets.

**Results:**

The main components of MYP include trehalose, morroniside, hypaconitine, loganin, quercetin, kaempferol, and other compounds. MYP treatment improved sperm count, sperm motility, and expression of T, E2, and FSH in OA bulls. Amino acid metabolomics analysis revealed that MYP treatment influenced 67 metabolites in comparison to the OA group. Among these, 47 amino acid metabolites were found to be upregulated, including Arginine, Phenylalanine, and Serine, among others. Conversely, 20 amino acid metabolites exhibited downregulation. The discovery of cysteine and methionine metabolism, glycine, serine, and threonine metabolism, alanine, aspartate, and glutamate metabolism, arginine biosynthesis, D-amino acid metabolism, the biosynthesis of phenylalanine, tyrosine, and tryptophan, as well as the mTOR signaling pathway, are significant metabolic pathways. Molecular docking results validated robust binding interactions between these active ingredients and their respective core targets.

**Conclusion:**

MYP exhibits significant therapeutic potential for OA in Simmental bulls by regulating hormone expression and restoring amino acid metabolic homeostasis. This present study elucidates the complex mechanisms through which MYP exerts its effects in the treatment of OA, thereby providing new evidence for understanding the pharmacological properties of traditional Chinese medicine for OA from multiple perspectives. Furthermore, MYP may represent a cost-effective therapeutic option for the treatment of OA in animals.

## 1 Introduction

Oligoasthenozoospermia (OA) is a pathological condition characterized by decreased sperm count and motility, representing a prevalent cause of male infertility (1). Sperm concentration, motility, and morphology are the most widely used diagnostic methods for male infertility (2). Modern medical research has concluded that oligozoospermia, which contributes to infertility in male animals, is a multifaceted disorder involving various factors such as reproductive tract infection, varicoceles, seminal plasma abnormalities, genetic diseases, immune diseases, idiopathic asthenospermia, endocrine diseases, environmental and physical factors, and other pathogenic elements (3). Additionally, lifestyle factors and metabolic disorders also impact sperm motility and have been increasingly recognized as significant contributors to impaired sperm motility (4). The pathological mechanisms underlying OA primarily include genetic factors, environmental factors, oxidative stress, infections and drug-induced damage (5). It is also associated with environmental pollution, long-term deficiencies in trace elements, unhealthy lifestyle habits, chronic mental stress, substance abuse (including drugs and hormone therapies), sexually transmitted diseases, and other contributing factors (53). Numerous empirical therapies exist for the treatment of OA, such as gonadotropins, anti-estrogens, aromatase inhibitors, and antioxidants, including vitamins, micronutrients, and carnitine (6). Clinical practice for managing oligospermia specifically involves the use of antioxidant drugs (e.g., L-carnitine), anti-infective agents, and steroid drugs. However, these treatments can be costly and may be accompanied by adverse side effects. Consequently, the development of traditional Chinese medicine formulas for treating OA holds significant promise.

The clinical application of herbal formulations in traditional Chinese veterinary medicine for male reproductive disorders has been documented for millennia (7). The “*JingYue QuanShu*” empirically categorized conditions analogous to oligoasthenozoospermia into phenotypic patterns, including kidney-yang deficiency, kidney-yin deficiency, and dual deficiency, descriptive classifications based on observable symptom clusters rather than modern pathophysiological mechanisms (8). Among traditional formulations, Yougui Pill (composed of *Aconitum napellus*, *Cinnamomum cassia*, Cuscuta chinensis, among others) was historically applied to cases presenting with yang deficiency-associated symptoms (9, 10). Notably, these phenotypic classifications served as heuristic frameworks for therapeutic development rather than mechanistic explanations. In this study, we developed Modified Yougui Powder (MYP) by removing the prohibitively expensive antler gum component from the traditional formula. Importantly, the investigation evaluates MYP’s effects on Simmental bulls’ oligoasthenospermia through contemporary biomedical parameters, with observed outcomes interpreted strictly within modern reproductive biology paradigms. The reference to historical TCM classifications serves solely to contextualize the formulation’s origins, not to imply dependency on traditional theoretical constructs for biological activity.

Some amino acids in semen are closely related to sperm motility (11). Various amino acids have cryoprotective effects during the freezing and thawing processes of mammalian sperm (12). Glutamic acid and proline can improve sperm motility, as well as the membrane and acrosomal integrity of frozen–thawed sperm (13). Phenylalanine has been successfully used as a therapeutic agent for oligospermia (11). The reduction of tryptophan leads to decreased sperm count in rats, which is associated with androgen excretion and receptor expression (14). Therefore, the present study combined the systematic strategies of UPLC-MS/MS and targeted amino acid metabolomics with MYP to explore the major compounds in MYP, the changes of amino acid metabolites in the semen of Simmental bulls, and potential metabolism pathways, providing theoretical references for the development and utilization of MYP.

## 2 Materials and methods

### 2.1 UPLC-MS/MS analysis of MYP

#### 2.1.1 Preparation of MYP

MYP decoction is prepared using the following ingredients: 6 g *Aconitum napellus*, 6 g cinnamon, 12 g dodder, 12 g *Eucommia ulmoides*, 9 g *Cornus officinalis*, 12 g Chinese yam, 9 g wolfberry, 24 g *Rehmannia glutinosa*, and 9 g *Angelica sinensis*. The preparation process entails mixing and grinding the ingredients, followed by filtration through four layers of 60-mesh gauze. Subsequently, water is added in a volume 10 times that of the crushed mixture. The mixture is soaked for 30 min and then boiled for 60 min. After boiling, the solution is filtered through four layers of gauze. To the filter residue, an additional eight times the amount of water is added and boiled again for another 60 min. This second extract is also filtered to obtain two separate filtrates, which are then combined. The combined filtrate undergoes centrifugation at a speed of 3,000 rpm for 5 min. Following this step, concentration under reduced pressure is performed using a rotary evaporator set at a temperature of 60°C and rotating at a speed of 60 rpm until it reaches a consistency suitable for hanging on the wall. Finally, vacuum freeze-drying is conducted to yield MYP decoction, which should be stored at low temperatures to maintain its efficacy.

#### 2.1.2 Pre-processing

Take the sample and grind it evenly with liquid nitrogen. Weigh approximately 100 mg of the sample into a 1.5 mL centrifuge tube. Add 1 mL of methanol–water solution (V: V = 3:1), which contains a mixed internal standard at a concentration of 4 μg/mL. Vortex for 1 min, then add steel balls to the mixture. Pre-cool in the refrigerator at −40°C for 2 min before placing it into the grinder for grinding at 60 Hz for 2 min. Subsequently, perform ultrasonic extraction in an ice water bath for 60 min, followed by standing at −40°C for an additional 30 min. Centrifuge the mixture for 10 min at a speed of 12,000 rpm and a temperature of 4°C. Dilute the resulting supernatant tenfold with methanol–water solution (V: V = 3:1) containing mixed internal standard (4 μg/mL). Finally, take out and analyze a volume of 200 μL from the supernatant.

#### 2.1.3 Ultra liquid chromaticity mass spectrometry conditions

The instrument utilized is a liquid chromatography-mass spectrometry system comprising an ACQUITY UPLC I-Class HF ultra-high performance liquid chromatography coupled with a QE high-resolution tandem mass spectrometer. Chromatographic conditions are as follows: Column ACQUITY UPLC HSS T3 (100 mm × 2.1 mm, 1.8 μm); Column temperature: 45°C; The mobile phase consisted of A-water (containing 0.1% formic acid) and B-acetonitrile. The flow rate was set at 0.35 mL/min, with an injection volume of 5 μL. The PDA scanning range spanned from 210 to 400 nm. The gradient elution procedure is outlined as follows: 0–2 min: 5% B; 2–4 min: increase from 5 to 30% B; 4–8 min: increase from 30 to 50% B; 8–10 min: increase from 50 to 80% B; 10–14 min: maintain at 80–100% B; 14–15 min: hold at a constant level of B at 100%; 15–15.1 min: decrease linearly from B at 100% to B at 5%; Maintain B at 5%for the durationof15–16 min.

Mass spectrometry conditions included an ion source operating in HESI mode, with sample mass spectrometry signals collected using both positive and negative ion scanning modes. The data collection was conducted in DDA scanning mode, specifically Full MS/d-MS2 (TOP 8). The parameters for mass spectrometry are detailed in Table 1.

**Table 1:** Mass spectrometry parameter information.

Parameter	Positive ion	Negative ion
Spray voltage (V)	3,800	−3000
Capillary temperature (°C)	320	320
Aux gas heater temperature (°C)	350	350
Sheath gas flow rate (Arb)	35	35
Aux gas flow rate (Arb)	8	8
S-lens RF level	50	50
Mass range (m/z)	100–1,200	100–1,200
Full MS resolution	70,000	70,000
MS/MS resolution	17,500	17,500
NCE/stepped NCE	10, 20, 40	10, 20, 40

### 2.2 Treatment of experimental animals

Taking Gansu Provincial Livestock Breeding and Improvement Management Station as the experimental base. This experimental study adhered to the relevant regulations concerning animal welfare and received approval from the Animal Experiment Ethics Committee of Gansu Agricultural University (GSAU-Eth-VMC-2023-051) prior to the implementation of the research plan. We selected 20 Simmental bulls aged between 3 and 6 years with similar body conformation and minimal size variations. Among these, 10 bulls were designated as the control group (with sperm count >5 × 10^7^/mL and sperm motility>50%; they were fed a basic diet). Clinically, the observed bulls exhibited scattered back hair, reduced libido, and weakness in the lumbar and pelvic regions. Artificial sperm collection was conducted daily from 8:00 a.m. to 10:00 a.m. A sample of 5 μL of original semen is taken for examination under a microscope at 400x magnification; if the sperm count is less than 5 × 10^7^/mL or if sperm motility is below 50%, one of these indicators confirms a diagnosis of OA in Simmental bulls (those fed with a basic diet supplemented with MYP). During the experiment, we will observe and record each bull’s mental state, coat glossiness, and any signs of curled-up or hunched-back posture. Semen samples will be collected and stored at −80°C.

#### 2.2.1 Preparation of MYP

Composition of MYP: 24 g *Aconitum napellus*, 24 g cinnamon, 48 g dodder, 48 g *Eucommia ulmoides*, 36 g *Cornus officinalis*, 48 g Chinese yam, 36 g wolfberry, 96 g *Rehmannia glutinosa*, and 36 g *Angelica sinensis*. All traditional Chinese medicines were sourced from the Yellow River Medicinal Materials Market in Lanzhou. The materials were subsequently crushed and blended into a coarse powder before being incorporated into bull feed and evenly mixed with concentrate. Each bull received one dose per day for 28 consecutive days. Throughout the experiment, bulls were ensured a normal intake of coarse feed and ad libitum water.

#### 2.2.2 Collection of bulls semen and semen quality testing

The artificial vagina method is used to stimulate the collection of bulls’ semen, and a semen collection cup is used to collect semen. Determine the color and odor of the collected semen, and discard semen that cannot meet the standards of “*Frozen Semen for bulls*.” Collect once before and after administration.

#### 2.2.3 Bulls blood collection

Following a 28-day feeding period, fasting blood samples were collected from the jugular vein of the bull on day 29. The blood was allowed to stand in a refrigerator at 4°C for 24 h and subsequently centrifuged at 3,000 rpm for 15 min at the same temperature. The supernatant (serum) was carefully extracted from the blood sample, leaving behind the residual plasma. After appropriate labeling, it was stored in a −80°C freezer for future analysis.

#### 2.2.4 Detection of serum hormones

Thaw the frozen serum designated for ELISA detection. Adhere strictly to the provided instructions and utilize the Beijing Huaying Biotechnology Research Institute ELISA kit to for the measurement of serum testosterone T (HY-C0007), estradiol E2 (HY-C0005), follicle-stimulating hormone FSH (HY-C0001), and luteinizing hormone LH (HY-C0002).

#### 2.2.5 Semen UHPLC-MRM-MS/MS target amino acid metabolomics detection

##### 2.2.5.1 Extraction of metabolites from semen and preparation of standard solutions

Thaw the semen in an ice water bath and vortex for 30 s to ensure thorough mixing before sampling. Accurately transfer 50 μL of the sample into a 1.5 mL EP tube using a pipette, then add 200 μL of extraction solution (acetonitrile: methanol, volume ratio 1:1, containing an isotope internal standard mixture, pre-cooled at −40°C). Vortex and mix for another 30 s. Subject the sample to ultrasound in an ice water bath for 15 min. Allow the sample solution to stand at −40°C for 1 h. Centrifuge the sample at 4°C and a speed of 12,000 rpm for 15 min. Carefully collect 100 μL of the supernatant and evaporate it to dryness using rotary evaporation. Dissolve the residue in 100 μL of a methanol–water mixture (50% methanol), then add 100 μL of derivatizing agent along with 50 μL of a sodium bicarbonate solution (1 mmol/L). Vortex thoroughly to mix well. Derivatize by incubating in a water bath at 40°C for 1 h; subsequently, remove from heat and allow cooling to room temperature. Add 50 μL of hydrochloric acid (2 mmol/L) and again evaporate to dryness via rotary evaporation. Finally, dissolve the dried residue in 200 μL of methanol and proceed with analysis on the machine.

Accurately weigh the appropriate amount of standard into a 10 mL volumetric flask to prepare a stock solution with a concentration of 10 mmol/L. Subsequently, take the corresponding volume of this standard stock solution and dilute it in another 10 mL volumetric flask to create a mixed standard solution. Sequentially dilute this mixed standard solution to generate a series of calibration solutions, which should include isotopically labeled internal standards at concentrations equivalent to those of the samples being analyzed.

##### 2.2.5.2 Mobile phase conditions

The target compounds were separated using a Thermo Vanquish UHPLC System (Thermo Fisher) equipped with ultra-high-performance liquid chromatography. The separation was performed on a Waters ACQUITY UPLC BEH C18 column (100 × 2.1 mm, 1.7 μm, United States). The mobile phases consisted of 5 mM ammonium acetate in phase A and acetonitrile in phase B. The column temperature was maintained at 45°C, while the sample tray was set to 4°C. An injection volume of 2 μL was utilized for the analysis.

Mass spectrometry analysis was conducted using the Thermo Altis TSQ Plus Mass Spectrometer, a triple quadrupole mass spectrometer equipped with an ESI electrospray ion source, operating in multi-reaction monitoring (MRM) mode. The parameters for the ion source were as follows: Spray Voltage = −3,300 V, Sheath Gas = 40 Arb, Aux Gas = 10 Arb, Sweep Gas = 1 Arb, Ion Transfer Tube Temperature = 325°C, Vaporizer Temperature = 350°C. Before conducting UHPLC–MS/MS analysis, a standard solution of the target compound was introduced into the mass spectrometer. For each target compound analyzed, several parent-daughter ion pairs (transitions) exhibiting the highest signal intensity were selected; their MRM parameters were optimized accordingly to identify the ion pair that provided the best response for quantitative analysis, while other ion pairs served for qualitative assessment of the target compound. All quantitative analyses of target compounds were performed using Skyline software, and data collection was executed via Xcalibur (version 4.4.16.14; Thermo Fisher).

##### 2.2.5.3 Calibration curve

Perform UPLC-MRM-MS/MS analysis on the calibration solution using method 2.3.5.2. Y represents the ratio of peak area between the target compound and the corresponding internal standard, and x represents the concentration of the target compound (nmol/L). When employing the least squares method for regression analysis with weights set to 1/x, optimal accuracy and correlation coefficient (R^2^) of the calibration solution are achieved. Calibration points will be excluded if the recovery rate for a given concentration exceeds the range of 80–120%.

##### 2.2.5.4 Method detection limit and quantification limit

The calibration solution was diluted by a factor of two, followed by UHPLC-MRM-MS analysis. The detection limit and quantification limit of the method were calculated based on their respective signal-to-noise ratios. The lowest limit of detection (LLOD) is defined as the concentration corresponding to a signal-to-noise ratio of 3, while the lowest limit of quantification (LLOQ) corresponds to a signal-to-noise ratio of 10, in accordance with US FDA guidelines for bioanalytical method validation.

##### 2.2.5.5 Precision and accuracy

The precision of quantitation was assessed as relative standard deviation (RSD), determined by injecting analytical replicates of a quality control (QC) sample. The accuracy of quantitation was evaluated based on the analytical recovery of this QC sample. Percent recovery was calculated using the formula [(mean observed concentration)/(spiked concentration)] × 100%.

### 2.3 Molecular docking

Active ingredients identified through UPLC-MS/MS analysis of MYP under section 2.2 were downloaded from TCMSP.^1^ Corresponding core ingredients were obtained via the PDB database.^2^ Core targets with relatively high comprehensive scores were screened in semen-targeted amino acid metabolomics under section 3.5, subsequently, we searched for mTOR protein structures corresponding to these core targets, downloading three-dimensional structures while prioritizing files with recent publication years and high resolution. These files were then imported into AutoDock Vina software for molecular docking and visualization operations.

### 2.4 Statistical analyses

In processing data for MYP analysis, the original data underwent baseline filtering, peak recognition, integration, retention time correction, peak alignment, and normalization using Progenesis QI v3.0 metabolomics processing software before pattern recognition during preprocessing stages. Metabolomics analysis utilized SIMCA software for principal component analysis (PCA) pattern recognition to evaluate significance in differences among groups. To enhance group separation and understanding further, supervised orthogonal partial least squares discriminant analysis (OPLS-DA) was employed. In the OPLS-DA model, metabolites with a Variable Importance in Projection (VIP) score greater than 1.0 are considered significant for the potential identification of samples. Differential metabolites were analyzed using MetaboAnalyst 5.0. Other data analysis and plotting were conducted using SPSS version 26.0 and GraphPad Prism version 10.0, with results expressed as mean ± standard deviation (SD). Comparisons between two or more groups were performed using T-tests and one-way analysis of variance (ANOVA). Statistical significance was established at a *p*-value less than 0.05.

## 3 Results

### 3.1 Quality control of main compounds in MYP

**Figure 1:**
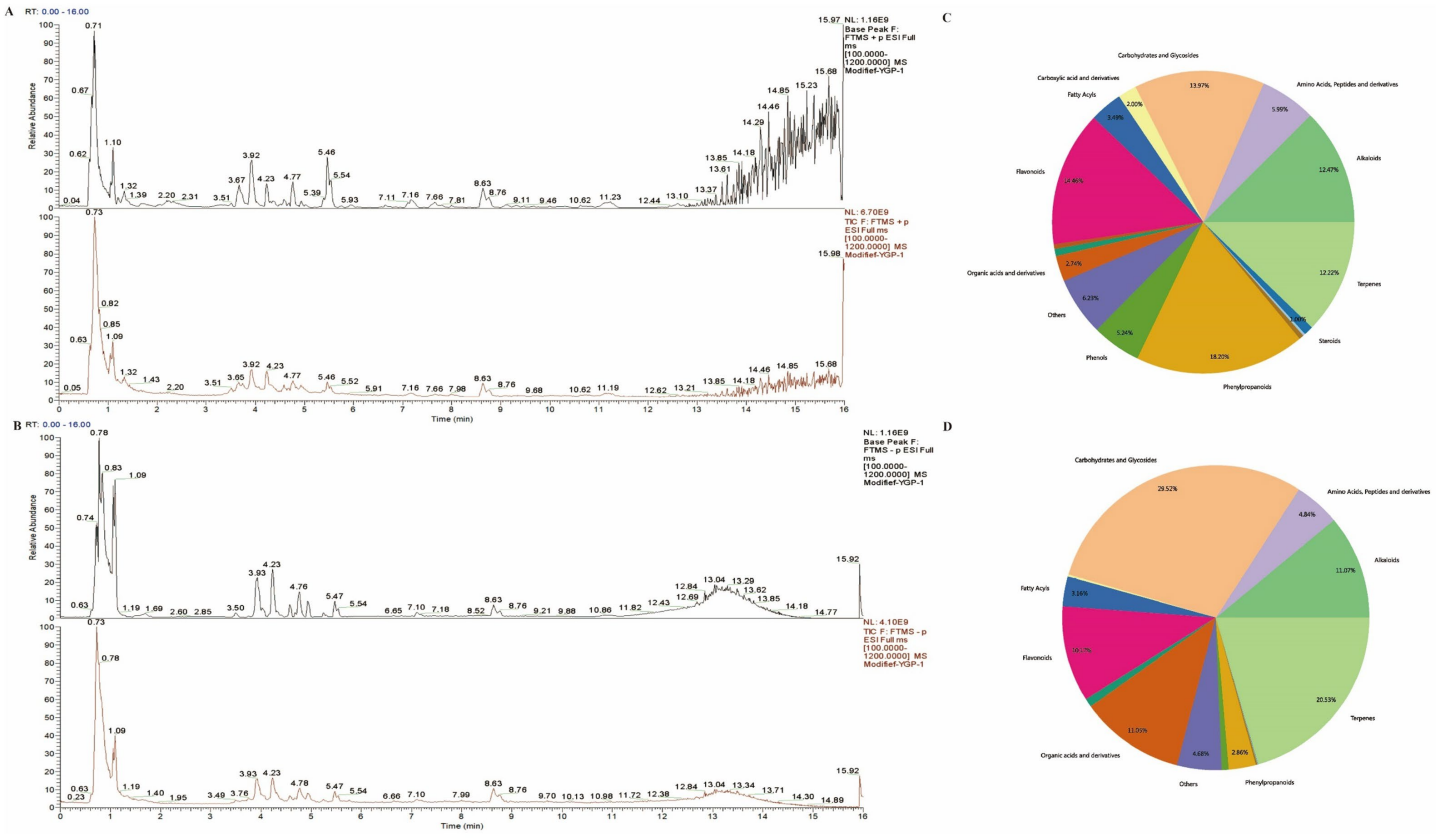
Schematic diagram illustrating total ion current in traditional Chinese medicine compounds (**A**: Positive ion mode; **B**: Negative ion mode). Classification of traditional Chinese medicine compounds (**C**: Content distribution diagram; **D**: Quantity distribution diagram).

The composition of MYP was analyzed utilizing ultra-high-performance liquid chromatography-mass spectrometry technology. A total of 315 compounds were detected in positive ion mode (POS), while 296 compounds were identified in negative ion mode (NEG). Total ion flux maps for MYP in both positive and negative modes are presented in Figures 1A,B. The classification regarding quantity and content distribution among traditional Chinese medicine ingredients is illustrated in Figures 1C,D. The five most effective compounds identified include trehalose (6.96%), morroniside (6.24%), maltotriose (5.49%), shanzhiside methyl ester (5.31%), and oxoglutaric acid (5.15%). Additionally, hypaconitine (2.88%), loganin (2.70%), adenosine (0.67%), gallic acid (0.27%), quercetin (0.06%), L-Arginine (0.05%), catalpol (0.05%), arbutin (0.03%), kaempferol (0 0.03%), L-Tryptophan (0 0.01%), along with other compounds were also detected as detailed in Table 2.

**Table 2:** Compounds identified within MYP (top 100).

Metabolites	Formula	Ion mode	Retention time (min)	Score	Mass error (ppm)	Theoretical m/z	Mean M (ratio of peak area %)
Trehalose	C_12_H_22_O_11_	POS	0.74	70.3	0.27	365.1054	6.964212615
Morroniside	C_17_H_26_O_11_	POS	3.92	57.1	0.14	429.1367	6.24388965
Maltotriose	C_18_H_32_O_16_	POS	0.92	63.9	0.72	527.1582	5.492068359
Shanzhiside methyl ester	C_17_H_26_O_11_	NEG	3.92	60.2	0.5	451.1457	5.312816983
Oxoglutaric acid	C_5_H_6_O_5_	NEG	0.78	56	−3.62	191.0199	5.150384187
7-[(beta-D-Glucopyranosyl)oxy]-3′,4′,5,8-tetrahydroxyflavone	C_21_H_20_O_12_	POS	4.77	64.6	0.27	465.1028	4.274130475
Diligustilide	C_24_H_28_O_4_	POS	11.16	52.5	0.22	403.188	4.244522721
Manninotriose	C_18_H_32_O_16_	NEG	0.67	58.6	0.25	549.1672	3.442857799
Citric acid	C_6_H_8_O_7_	NEG	1.09	67.5	−2.98	191.0197	3.41158771
Alpha-Lactose	C_12_H_22_O_11_	NEG	0.73	59.1	0.44	387.1144	3.382696734
Luciferin	C_11_H_8_N_2_O_3_S_2_	POS	5.46	60	−0.12	281.0049	2.880440326
Hypaconitine	C_33_H_45_NO_10_	POS	7.18	59.1	0.45	616.3116	2.707928379
Loganin	C_17_H_26_O_10_	POS	4.23	41.9	−0.1	413.1418	2.144899385
Benzoylmesaconine	C_31_H_43_NO_10_	POS	5.39	52.4	0.18	590.296	2.011129202
Quercetin 3-sambubioside	C_26_H_28_O_16_	NEG	4.57	63.1	0.64	595.1305	1.858736874
Melibiose	C_12_H_22_O_11_	POS	0.73	69.4	0.18	365.1054	1.71377763
13-Docosenamide	C_22_H_43_NO	POS	14.89	65.5	−0.44	338.3417	1.506990262
Sesamose	C_24_H_42_O_21_	POS	0.89	64.4	0.57	689.2111	1.255961741
L-Proline	C_5_H_9_NO_2_	POS	0.74	56.9	2.65	116.0706	1.255771959
Gluconic acid	C_6_H_12_O_7_	NEG	0.72	58.8	−2.79	195.051	1.211085242
Cornuside	C_24_H_30_O_14_	POS	4.93	50.2	0.41	565.1528	1.193618311
Neochlorogenic acid	C_16_H_18_O_9_	POS	4.03	57	0.26	377.0843	1.056336274
Melezitose	C_18_H_32_O_16_	POS	0.79	54.6	0.61	522.2028	0.926595897
Benzoylhypaconine	C_31_H_43_NO_9_	POS	5.94	55.2	0.71	574.3011	0.794956317
Verbascoside	C_29_H_36_O_15_	NEG	4.81	58	0.73	623.1981	0.751668325
Forsythoside H	C_29_H_36_O_15_	NEG	4.68	61.1	0.69	623.1981	0.73826844
Glucose	C_6_H_12_O_6_	NEG	0.7	57.5	−1.72	225.0617	0.711673167
Neocretanin	C_20_H_20_O_13_	NEG	3.9	53.6	0.53	513.0886	0.700028938
Pyroglutamic acid	C_5_H_7_NO_3_	POS	1.1	57.9	1.45	130.0499	0.681427151
Adenosine	C_10_H_13_N_5_O_4_	POS	1.22	60.8	0.39	268.104	0.675280526
Oleamide	C_18_H_35_NO	POS	12.62	65.8	−0.05	282.2791	0.650920518
Isomaltotetraose	C_24_H_42_O_21_	NEG	0.67	56.8	0.86	711.22	0.642801109
Neoline	C_24_H_39_NO_6_	POS	4.36	53.9	0.52	438.285	0.614868108
Gardaloside	C_16_H_22_O_9_	NEG	4.3	58.3	0.49	403.1246	0.564884776
Benzoylaconine	C_32_H_45_NO_10_	POS	5.75	51.3	0.69	604.3116	0.55193389
Mesaconitine	C_33_H_45_NO_11_	POS	6.57	58.4	0.67	632.3065	0.422287999
Quercetin 3-laminaribioside	C_27_H_30_O_17_	POS	3.93	59.7	0.76	627.1556	0.414686045
Senkyunolide I	C_12_H_16_O_4_	POS	5.55	55.6	0.26	247.0941	0.414197395
Fuziline	C_24_H_39_NO_7_	POS	4.25	58.4	0.28	454.2799	0.381322221
Arginine	C_6_H_14_N_4_O_2_	POS	0.68	51.3	0.4	213.0748	0.347311915
Rosiridin	C_16_H_28_O_7_	POS	5.03	51	0.14	355.1727	0.322011053
L-Isoleucine	C_6_H_13_NO_2_	POS	1.34	57.3	1.41	132.1019	0.316754127
D-Arabonate	C_5_H_10_O_6_	NEG	0.72	56.5	−4.25	165.0405	0.302984193
D-Pipecolic acid	C_6_H_11_NO_2_	POS	0.78	52.9	1.49	130.0863	0.297695576
Nepitrin	C_22_H_22_O_12_	POS	5	61.4	0.48	479.1184	0.290583729
Trifolin	C_21_H_20_O_11_	POS	4.97	65.3	0.41	449.1078	0.290441726
Talatisamine	C_24_H_39_NO_5_	POS	4.54	61.4	0.36	422.2901	0.282009861
Gallic acid	C_7_H_6_O_5_	NEG	1.69	54.9	−3.46	169.0142	0.272265803
Lucidin 3-o-beta-primveroside	C_26_H_28_O_14_	NEG	4.94	42.2	−3.5	609.1463	0.270237584
3,6-anhydrogalactose	C_6_H_10_O_5_	NEG	0.77	61	−2.49	207.0511	0.256285746
2-O-beta-D-Glucopyranosyl-L-ascorbic acid	C_12_H_18_O_11_	NEG	1.04	55.3	0.44	337.0776	0.254916194
Tagatose	C_6_H_12_O_6_	NEG	0.8	63.9	−3.8	161.0455	0.24044178
L-Phenylalanine	C_9_H_11_NO_2_	POS	2.06	59.3	0.95	166.0863	0.232432443
Ascorbyl glucoside	C_12_H_18_O_11_	POS	1.05	48.5	0.1	361.0741	0.231340141
Robinetin	C_15_H_10_O_7_	POS	4.77	51.2	−0.37	303.0499	0.224530424
Quercetin 3-o-neohesperidoside	C_27_H_30_O_16_	NEG	4.63	76.7	0.9	609.1461	0.22326303
Aconitine	C_34_H_47_NO_11_	POS	7.14	58.3	0.62	646.3222	0.213474371
dehydroascorbates	C_6_H_6_O_6_	NEG	0.8	50.8	−3.62	173.0092	0.21049324
Magnoloside B	C_35_H_46_O_20_	NEG	4.21	59	0.53	785.251	0.190708142
4-Hydroxy-2-methoxybenzoicacid	C_8_H_8_O_4_	NEG	4.2	60.2	−3.81	167.035	0.18958849
Swertiamacroside	C_21_H_28_O_13_	NEG	4.16	51.2	0.7	487.1457	0.184656059
Acetoin glucoside	C_10_H_20_O_7_	NEG	1.4	55.6	0.57	297.1191	0.184110716
Quebrachitol	C_7_H_14_O_6_	POS	0.82	40.5	0.63	217.0682	0.182344251
N-(1-Deoxy-1-fructosyl)tyrosine	C_15_H_21_NO_8_	POS	1.11	53.4	0.62	344.134	0.180224711
Triptocalline A	C_28_H_42_O_4_	POS	10.74	54.8	−3.3	481.2716	0.179687101
N-(1-Deoxy-1-fructosyl)phenylalanine	C_15_H_21_NO_7_	POS	2.08	53.4	0.37	328.1391	0.174696855

### 3.2 MYP treatment can improve sperm quality of OA in Simmental bulls

**Figure 2:**
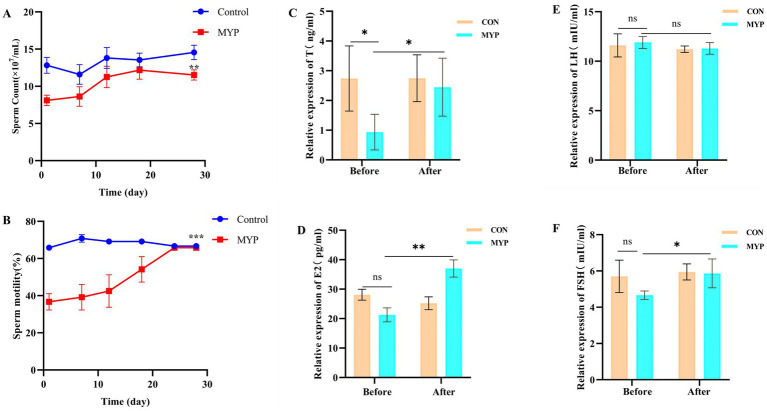
Sperm quality and hormone levels. **p < 0.01, ***p < 0.001 compared with the first day in **(A,B)**. *p < 0.05, **p < 0.01, ^ns^ *p* > 0.05 compared with the Control group in **(C–F)**.

The semen color of Simmental bulls is visibly milky white and exhibits a slight fishy odor. The control group demonstrated stable sperm count and motility, whereas the MYP group exhibited an upward trend following treatment, ultimately reaching normal levels after 30 days (Figures 2A,B). MYP has shown significant therapeutic effects on OA in Simmental bulls and can enhance semen quality.

### 3.3 The effect of MYP treatment on hormones of OA in Simmental bulls

The levels of T, E2, FSH, and LH in the serum before and after administration were detected using an ELISA kit in Simmental bulls. The results showed that the levels of T, E2, and FSH in the serum after administration significantly increased and reached the level of the control group (*p* < 0.05). The E2 content significantly increased (*p* < 0.01), while the LH content did not show significant changes (Figures 2C–F). The above results indicate that MYP improved the decrease in hormone levels in OA. Since the control group did not show significant changes before and after treatment, the subsequent experiments observed the changes before and after treatment in the MYP group.

### 3.4 Amino acid targeted metabolomics analysis

#### 3.4.1 Metabolic analysis of semen samples

**Figure 3:**
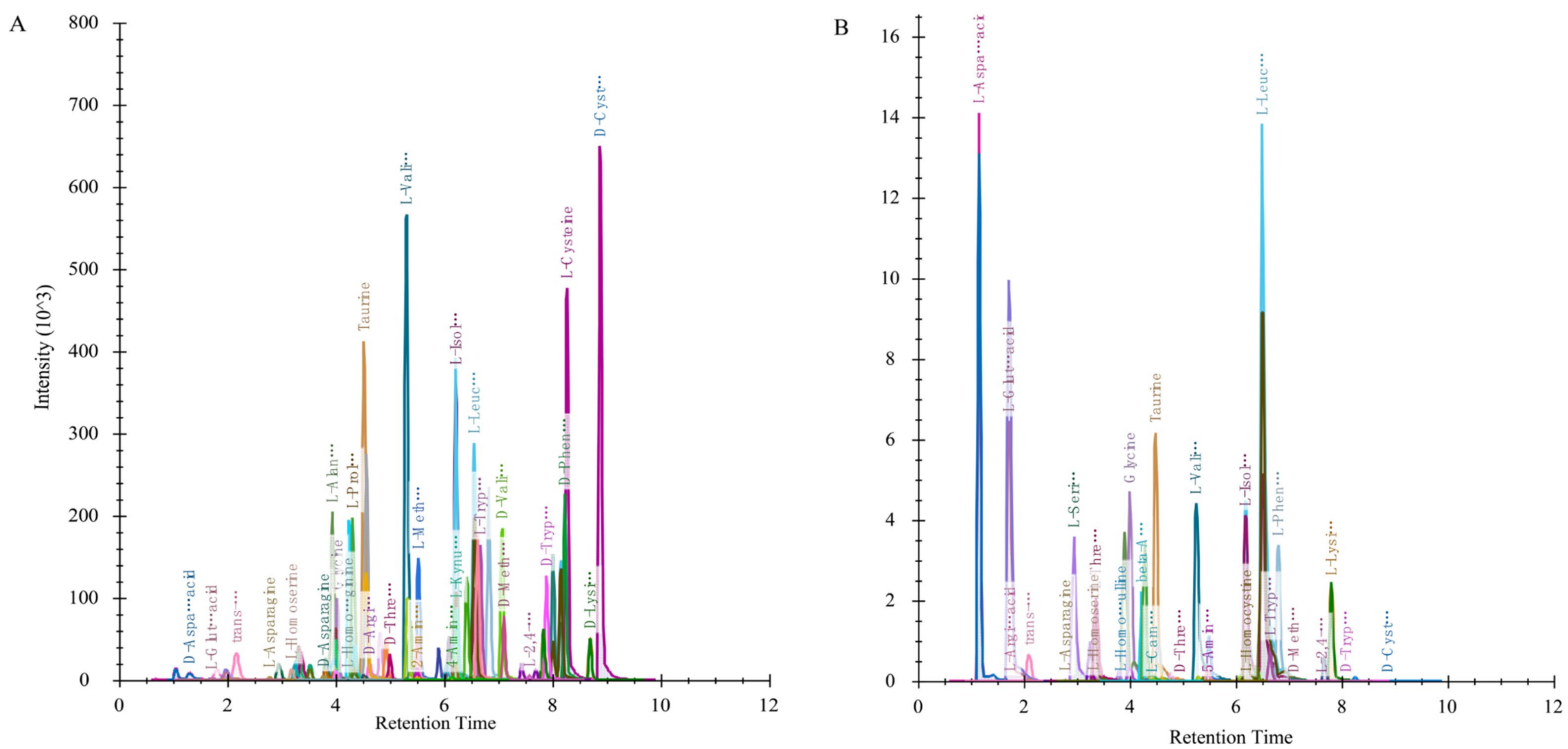
Example of ion chromatogram extracted from some indicators. Ionic chromatograms extracted from standard solution **(A)** and sample **(B)**.

The analytical method employed in this experiment demonstrated that all target compounds exhibited symmetrical chromatographic peaks, successfully achieving the chromatographic separation of the target compounds. There were no significant differences observed in the retention time and chromatographic peak shape of the target compounds between biological samples and standard solutions (Figure 3). The lowest detection limits (LLODs) for each target compound ranged from 0.31 to 312.50 nmol/L, while the lowest quantification limits (LLOQs) varied between 0.61 and 625.00 nmol/L. The correlation coefficients (R^2^) exceeded 0.9801, indicating a robust quantitative relationship between chromatographic peak area and compound concentration, thereby fulfilling the requirements for targeted metabolomics analysis. Quality control (QC) samples were injected five times repetitively, yielding an average recovery rate for all target compounds ranging from 80.8 to 119.0%, with standard relative deviations remaining below 15.1%. This method can accurately and reliably quantify the content of target metabolites within the aforementioned concentration range (Table 3).

**Table 3:** Content analysis of target metabolites in samples.

Compound name	LLOD (nmol/L)	LLOQ (nmol/L)	ULOQ (nmol/L)	R^2^
1-Methyl-L-histidine	2.44	4.88	10000.00	0.9877
2-Aminoisobutyric acid	2.44	4.88	10000.00	0.9909
3-Methyl-histidine	4.88	9.77	10000.00	0.9825
4-Aminobenzoic acid	19.53	39.06	2500.00	0.9841
5-Aminovaleric acid	0.31	0.61	5000.00	0.9841
D-Alanine	0.61	1.22	10000.00	0.9868
D-Arginine	4.88	9.77	10000.00	0.9900
D-Asparagine	0.61	1.22	10000.00	0.9917
D-Aspartic acid	312.50	625.00	10000.00	0.9963
D-Citrulline	0.61	1.22	10000.00	0.9914
D-Cysteine	0.31	0.61	10000.00	0.9883
D-Glutamic acid	9.77	19.53	10000.00	0.9846
D-Glutamine	2.44	4.88	625.00	0.9972
D-Histidine	0.61	1.22	5000.00	0.9894
D-Isoleucine	0.31	0.61	10000.00	0.9916
D-Lysine	0.31	0.61	10000.00	0.9822
D-Methionine	0.61	1.22	10000.00	0.9807
D-Ornithine	0.61	1.22	10000.00	0.9900
D-Phenylalanine	0.31	0.61	2500.00	0.9816
D-Proline	0.61	1.22	5000.00	0.9920
D-Serine	0.61	1.22	2500.00	0.9818
D-Threonine	0.61	1.22	10000.00	0.9994
D-Tryptophan	0.61	1.22	10000.00	0.9806
D-Tyrosine	0.31	0.61	5000.00	0.9805
D-Valine	0.31	0.61	10000.00	0.9870
D-leucine	0.31	0.61	312.50	0.9929
Ethanolamine	4.88	9.77	20000.00	0.9886
Glutathione	1.22	2.44	2500.00	0.9891
Glycine	312.50	625.00	10000.00	0.9933
L-2,4-diaminobutyric acid	1.22	2.44	10000.00	0.9921
L-2-Aminoadipic acid	1.22	2.44	10000.00	0.9986
L-2-Aminobutyric acid	0.61	1.22	2500.00	0.9856
L-Alanine	2.44	4.88	10000.00	0.9802
L-Arginine	39.06	78.13	2500.00	0.9802
L-Argininosuccinic acid	0.31	0.61	312.50	0.9941
L-Asparagine	19.53	39.06	10000.00	0.9801
L-Aspartic acid	156.25	312.50	10000.00	0.9889
L-Carnosine	1.22	2.44	10000.00	0.9821
L-Citrulline	4.88	9.77	10000.00	0.9929
L-Cysteine	0.31	0.61	2500.00	0.9826
L-Cystine	0.31	0.61	1250.00	0.9848
L-Glutamic acid	2.44	4.88	156.25	0.9930
L-Glutamine	2.44	4.88	10000.00	0.9894
L-Histidine	0.31	0.61	10000.00	0.9940
L-Homoarginine	19.53	39.06	2500.00	0.9893
L-Homocitrulline	39.06	78.13	10000.00	0.9809
L-Homocystine	0.31	0.61	5000.00	0.9812
L-Homoserine	4.88	9.77	1250.00	0.9806
L-Isoleucine	0.31	0.61	5000.00	0.9899
L-Kynurenine	0.31	0.61	5000.00	0.9896
L-Leucine	0.31	0.61	2500.00	0.9921
L-Lysine	1.22	2.44	20000.00	0.9862
L-Methionine	0.61	1.22	10000.00	0.9939
L-Ornithine	156.25	312.50	10000.00	0.9818
L-Phenylalanine	2.44	4.88	10000.00	0.9864
L-Pipecolic acid	4.88	9.77	10000.00	0.9829
L-Proline	0.61	1.22	20000.00	0.9888
L-Serine	4.88	9.77	10000.00	0.9902
L-Threonine	0.31	0.61	5000.00	0.9956
L-Tryptophan	0.61	1.22	10000.00	0.9891
L-Tyrosine	1.22	2.44	5000.00	0.9896
L-Valine	0.61	1.22	5000.00	0.9905
L-γ-glutamyl-L-cysteine	0.61	1.22	2500.00	0.9867
Symmetric dimethylarginine	19.53	39.06	1250.00	0.9836
Taurine	39.06	78.13	10000.00	0.9825
trans-L-Hydroxyproline	0.31	0.61	10000.00	0.9903
β-Alanine	2.44	4.88	10000.00	0.9856
γ-Aminobutyric acid	4.88	9.77	10000.00	0.9972

**Figure 4:**
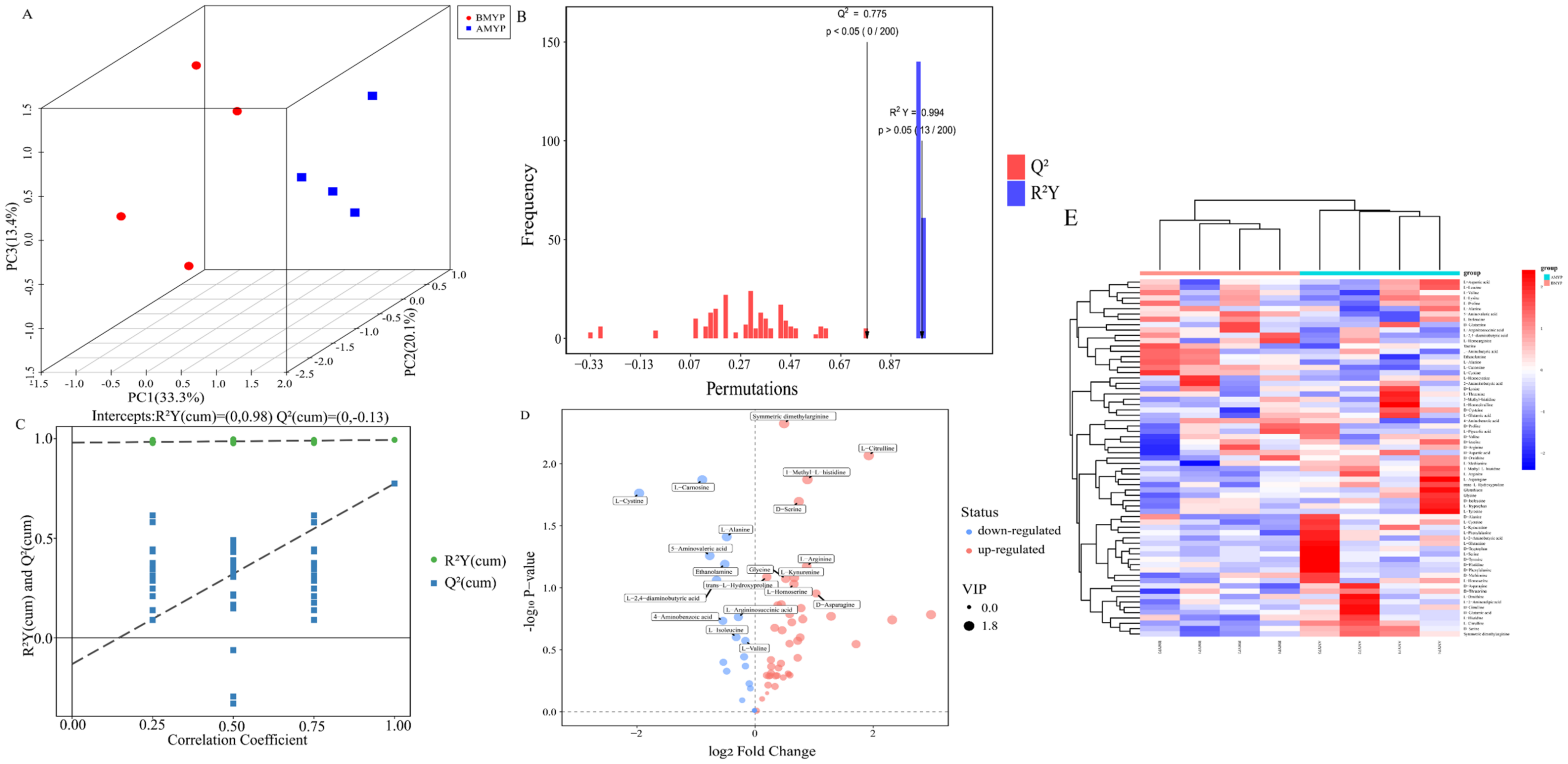
**(A)** PCA score scatter plot. **(B)** Bar chart depicting permutation test results for OPLS-DA model. **(C)** The permutation test results of OPLS-DA model. **(D)** 3D volcano map. **(E)** Hierarchical clustering analysis heatmap.

**Figure 5:**
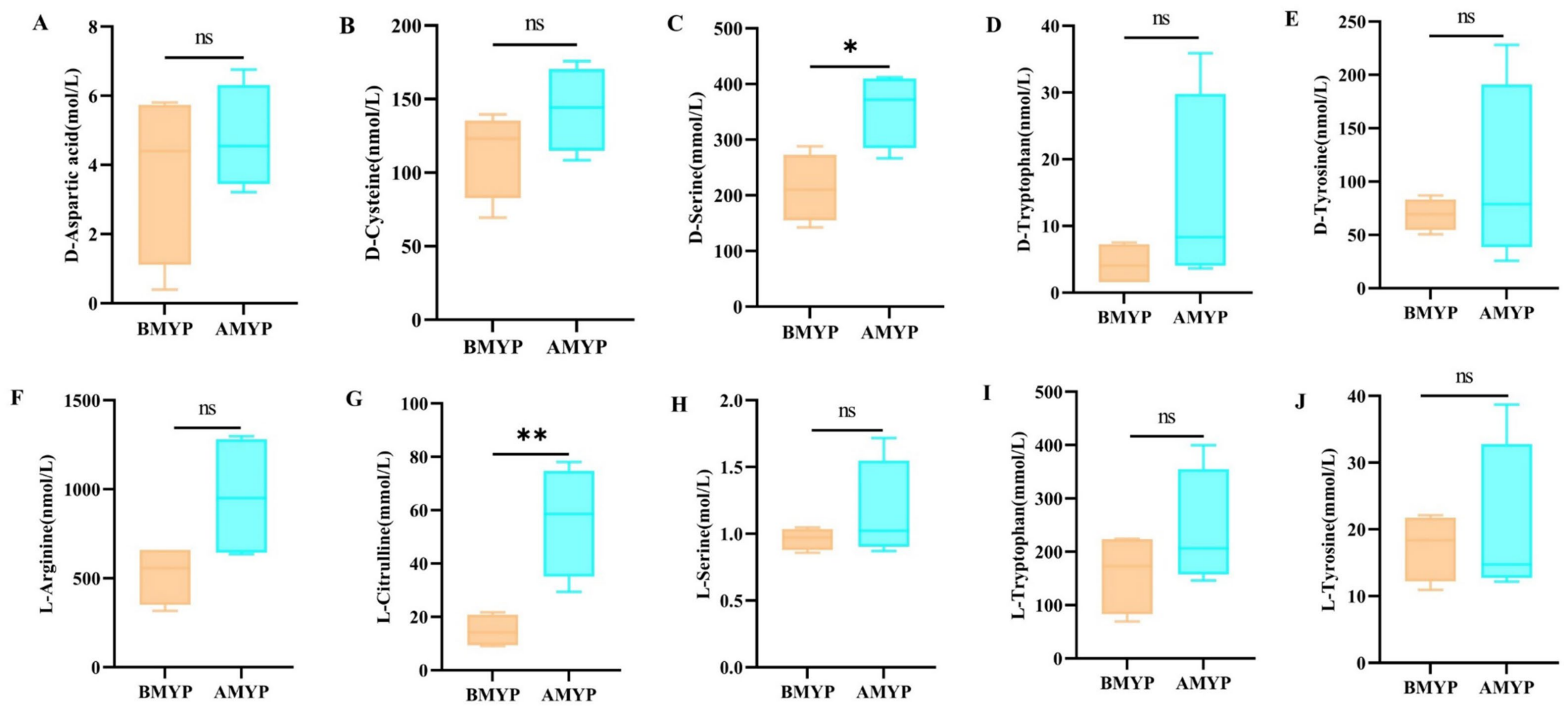
Changes in amino acid content (*p < 0.05, **p < 0.01, ^ns^*p* > 0.05 compared with the BMYP group. **A**: D-Aspartic acid; **B**: D-Cysteine; **C**: D-Serine; **D**: D-Tryptophan; **E**: D-Tyrosine; **F**: L-Arginine; **G**: L-Citrulline; **H**: L-Serine; **I**: L-Tryptophan; **J**: L-Tyrosine).

Principal Component Analysis (PCA) revealed a distinct separation between the before and after administration (BMYP) group and the after administration (AMYP) group (Figure 4A). A supervised multidimensional statistical approach known as Partial Least Squares Discriminant Analysis (PLS-DA) was utilized to analyze samples from both groups, resulting in model quality parameters: Accuracy = 1.0, *R*^2^ = 0.9985, *Q*^2^ = 0.88227. As illustrated in Figures 4B,C, both groups displayed excellent separability characterized by high R^2^Y and Q^2^ values. Through amino acid-targeted metabolomics analysis, a total of 67 differential amino acid metabolites were identified (Table 4), comprising 47 upregulated amino acids and 20 downregulated amino acids (Figure 4D). A heatmap was generated to visualize metabolic changes between these two groups (Figure 4E). Notably, D-aspartic acid, D-cysteine, D-serine, D-tryptophan, D-tyrosine, L-arginine, L-citrulline, L-serine, L-tryptophan, and L-tyrosine exhibited upward trends. With statistically significant differences noted at *p* < 0.05 between D-serine and L-citrulline (Figure 5).

**Table 4:** Amino acid differential metabolites.

Compound name	M. W.	CAS	Pubchem	HMDB ID	Formula
Ethanolamine	61.08	141–43-5	700	HMDB0000149	C_2_H_7_NO
Glycine	75.07	56–40-6	750	HMDB0000123	C_2_H_5_NO_2_
D-Alanine	89.09	338–69-2	71,080	HMDB0001310	C_3_H_7_NO_2_
beta-Alanine	89.09	107–95-9	239	HMDB0000056	C_3_H_7_NO_2_
L-Alanine	89.09	56–41-7	5,950	HMDB0000161	C_3_H_7_NO_2_
2-Aminoisobutyric acid	103.12	62–57-7	6,119	HMDB0001906	C_4_H_9_NO_2_
L-2-Aminobutyric acid	103.12	1,492-24-6	80,283	HMDB0000452	C4H9NO2
gamma-Aminobutyric acid	103.12	56–12-2	119	HMDB0000112	C4H9NO2
L-Serine	105.09	56–45-1	5,951	HMDB0000187	C3H7NO3
D-Serine	105.09	312–84-5	71,077	HMDB0003406	C3H7NO3
L-Proline	115.13	147–85-3	145,742	HMDB0000162	C5H9NO2
D-Proline	115.13	344–25-2	8,988	HMDB0003411	C5H9NO2
5-Aminovaleric acid	117.15	660–88-8	138	HMDB0003355	C5H11NO2
L-Valine	117.15	72–18-4	6,287	HMDB0000883	C5H11NO2
D-Valine	117.15	640–68-6	71,563	HMDB0250806	C5H11NO2
D-Threonine	119.12	632–20-2	69,435	HMDB0250801	C4H9NO3
L-Threonine	119.12	72–19-5	6,288	HMDB0000167	C4H9NO3
L-Homoserine	119.12	672–15-1	12,647	HMDB0000719	C4H9NO3
Taurine	125.15	107–35-7	1,123	HMDB0000251	C2H7NO3S
L-Pipecolic acid	129.16	3,105-95-1	439,227	HMDB0000716	C6H11NO2
trans-L-Hydroxyproline	131.13	51–35-4	5,810	HMDB0000725	C5H9NO3
D-Isoleucine	131.17	319–78-8	76,551		C6H13NO2
L-Isoleucine	131.17	73–32-5	6,306	HMDB0000172	C6H13NO2
D-leucine	131.17	328–38-1	439,524	HMDB0013773	C6H13NO2
L-Leucine	131.17	61–90-5	6,106	HMDB0000687	C6H13NO2
D-Asparagine	132.12	2058-58-4	439,600	HMDB0033780	C4H8N2O3
L-Asparagine	132.12	70–47-3	6,267	HMDB0000168	C4H8N2O3
D-Aspartic acid	133.10	1783-96-6	83,887	HMDB0006483	C4H7NO4
L-Aspartic acid	133.10	56–84-8	5,960	HMDB0000191	C4H7NO4
4-Aminobenzoic acid	137.14	150–13-0	978	HMDB0001392	C7H7NO2
D-Glutamine	146.14	5,959-95-5	145,815	HMDB0003423	C5H10N2O3
L-Glutamine	146.14	56–85-9	5,961	HMDB0000641	C5H10N2O3
D-Glutamic acid	147.13	6,893-26-1	23,327	HMDB0003339	C5H9NO4
L-Glutamic acid	147.13	56–86-0	33,032	HMDB0000148	C5H_9_NO4
L-Methionine	149.21	63–68-3	6,137	HMDB0000696	C5H11NO2S
D-Methionine	149.21	348–67-4	84,815		C5H11NO2S
L-Histidine	155.15	71–00-1	6,274	HMDB0000177	C6H9N3O2
D-Histidine	155.15	351–50-8	71,083	HMDB0250763	C6H9N3O2
L-2-Aminoadipic acid	161.16	1,118-90-7	92,136	HMDB0000510	C6H11NO4
L-Phenylalanine	165.19	63–91-2	6,140	HMDB0000159	C9H11NO2
D-Phenylalanine	165.19	673–06-3	71,567	HMDB0250791	C9H11NO2
3-Methyl-histidine	169.18	368–16-1	64,969	HMDB0000479	C7H11N3O2
1-Methyl-L-histidine	169.18	332–80-9	92,105	HMDB0000001	C7H11N3O2
D-Arginine	174.20	157–06-2	71,070	HMDB0003416	C6H14N4O2
L-Arginine	174.20	74–79-3	6,322	HMDB0000517	C6H14N4O2
D-Citrulline	175.19	13,594–51-9	637,599	HMDB0250742	C6H13N3O3
L-Citrulline	175.19	372–75-8	9,750	HMDB0000904	C6H13N3O3
D-Tyrosine	181.19	556–02-5	71,098	HMDB0250803	C9H11NO3
L-Tyrosine	181.19	60–18-4	6,057	HMDB0000158	C9H11NO3
L-Homoarginine	188.23	156–86-5	9,085	HMDB0000670	C7H16N4O2
L-Homocitrulline	189.21	1,190–49-4	65,072	HMDB0000679	C7H15N3O3
Symmetric dimethylarginine	202.25	30,344–00-4	169,148	HMDB0003334	C8H18N4O2
D-Tryptophan	204.22	153–94-6	9,060	HMDB0013609	C11H12N2O2
L-Tryptophan	204.22	73–22-3	6,305	HMDB0000929	C11H12N2O2
L-Kynurenine	208.21	2,922-83-0	846	HMDB0000684	C10H12N2O3
L-Carnosine	226.23	305–84-0	439,224	HMDB0000033	C9H14N4O3
L-Argininosuccinic acid	290.27	2,387-71-5	16,950	HMDB0000052	C10H18N4O6
L-2,4-diaminobutyric acid	118.13	1758–80-1	134,490	HMDB0006284	C4H10N2O2
D-Cysteine	121.16	921–01-7	92,851	HMDB0003417	C3H7NO2S
L-Cysteine	121.16	52–90-4	5,862	HMDB0000574	C3H7NO2S
D-Ornithine	132.16	348–66-3	71,082	HMDB0003374	C5H12N2O2
L-Ornithine	132.16	70–26-8	6,262	HMDB0000214	C5H12N2O2
D-Lysine	146.19	923–27-3	57,449	HMDB0003405	C6H14N2O2
L-Lysine	146.19	56–87-1	5,962	HMDB0000182	C6H14N2O2
L-Cystine	240.30	56–89-3	67,678	HMDB0000192	C_6_H_12_N2O4S2
L-gamma-glutamyl-L-cysteine	250.27	636–58-8	23,615,402	HMDB0001049	C_8_H_14_N_2_O_5_S
L-Homocystine	268.35	626–72-2	439,579	HMDB0000676	C_8_H_16_N_2_O_4_S_2_
Glutathione	307.33	70–18-8	124,886	HMDB0000125	C_10_H_17_N_3_O6S

#### 3.4.2 Metabolic pathway analysis

**Figure 6:**
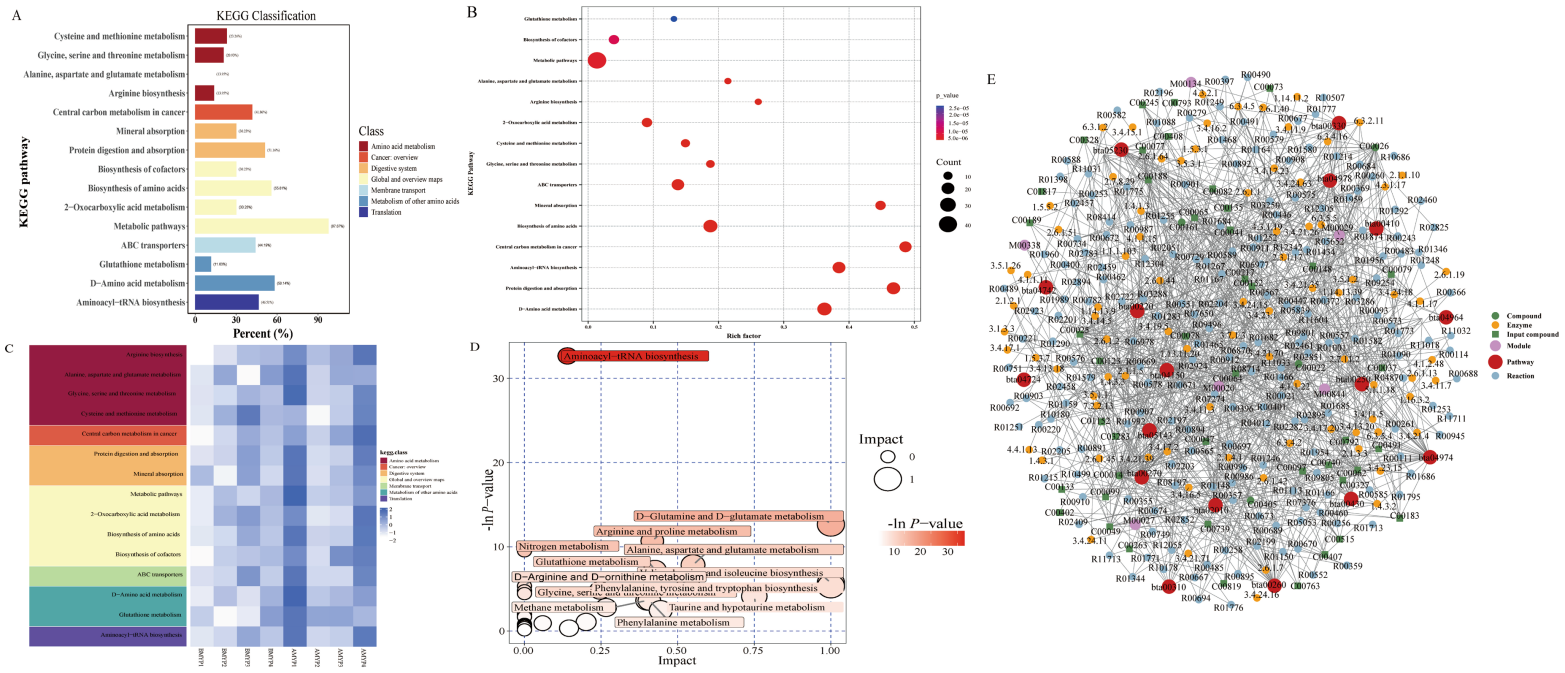
**A**: Differential metabolite KEGG classification diagram. **B**: Differential metabolite KEGG enrichment diagram. **C**: KEGG heatmap pathway analysis diagram. **D**: Pathway analysis diagram. **F**: Regulatory Network Analysis Diagram (Red dots represent metabolic pathway, yellow dots indicate information regarding substance-related regulatory enzymes, green dots denote background substances within a metabolic pathway, purple dots provide information about molecular modules related to specific substances, blue dots illustrate chemical interaction reactions among substances, and green squares signify differential substances identified through this comparison).

After MYP treatment, the majority of amino acid metabolites were restored, indicating a reduction in metabolic disturbances. To further explore the metabolic pathways regulated by MYP, differential metabolites were analyzed using MetaboAnalyst 5.0. Fifteen metabolic pathways exhibited restoration following MYP treatment based on pathway effects greater than 0.1. These pathways include cysteine and methionine metabolism; glycine, serine, and threonine metabolism; alanine, aspartate, and glutamate metabolism; arginine biosynthesis; D-amino acid metabolism; as well as the biosynthesis of phenylalanine, tyrosine, and tryptophan (Figure 6).

#### 3.4.3 Metabolite regulatory network analysis

Based on the differential metabolites identified from previous analyses, a network enrichment analysis was conducted. The results encompassed various metabolic pathways such as arginine biosynthesis; alanine, aspartate, and glutamate metabolism; glycine, serine, and threonine metabolism; cysteine and methionine metabolism, among others, including the mTOR signaling pathway. Following the acquisition of matching information for differential metabolites across each group, pathway searches and regulatory interaction network analyses were performed utilizing the corresponding *Bos taurus* (bull) KEGG database. The outcomes of this regulatory analysis are presented in a network plot (Figure 6F), while an overview of selected differential metabolite regulatory networks is provided in Table 5.

**Table 5:** Analysis of differential metabolite regulatory networks.

KKEGG.id	Entry. type	KEGG.name	p.score
bta00220	Pathway	Arginine biosynthesis	6.8E-06
bta00250	Pathway	Alanine, aspartate and glutamate metabolism	0.000001
bta00260	Pathway	Glycine, serine, and threonine metabolism	0.000001
bta00270	Pathway	Cysteine and methionine metabolism	0.000001
bta04150	Pathway	mTOR signaling pathway	0.000001
M00020	Module	Serine biosynthesis, glycerate-3P = > serine	0.000001
M00027	Module	GABA (gamma-aminobutyrate) shunt	0.000001
M00029	Module	Urea cycle	2.13E-05
M00134	Module	Polyamine biosynthesis, arginine = > ornithine	0.000001
M00338	Module	Cysteine biosynthesis, homocysteine + serine	0.000001
1.1.1.103	Enzyme	L-threonine 3-dehydrogenase	0.000001
1.13.11.20	Enzyme	cysteine dioxygenase	1.14E-05
1.14.11.2	Enzyme	procollagen-proline 4-dioxygenase	0.000001
1.14.13.39	Enzyme	nitric-oxide synthase (NADPH)	0.000001
1.14.13.9	Enzyme	kynurenine 3-monooxygenase	3.46E-06
R00021	Reaction	L-glutamate:ferredoxin oxidoreductase	0.000001
R00093	Reaction	L-glutamate: NAD + oxidoreductase	0.000001
R00111	Reaction	N-(omega)-Hydroxyarginine, NADPH:oxygen oxidor	0.000001
R00114	Reaction	L-glutamate: NADP+ oxidoreductase	0.000001
R00220	Reaction	L-serine ammonia-lyase	0.000001
C00014	Compound	Ammonia	0.000001
C00022	Compound	Pyruvate	0.000441
C00025	Compound	L-Glutamate	0.000001
C00026	Compound	2-Oxoglutarate	0.001688
C00037	Compound	Glycine	0.000001

### 3.5 Molecular docking

**Figure 7:**
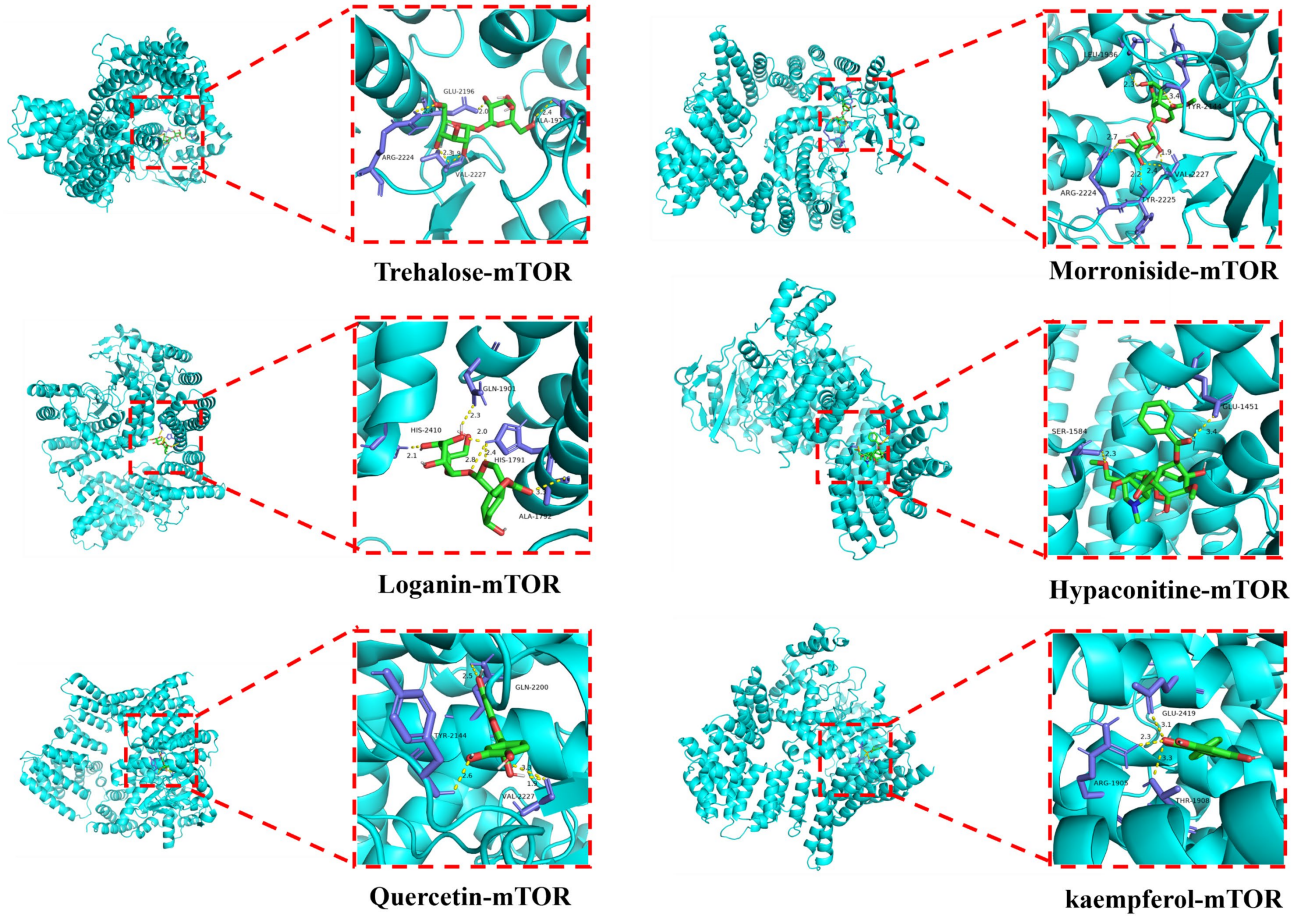
Docking mode between bioactive ingredients and core target molecules (Trehalose, morroniside, loganin, hypaconitine, quercetin, and kaempferol dock with mTOR).

Based on the results of UPLC-MS/MS analysis, alginose was selected for a docking study along with morroniside, strychnoside, hypaconitine, quercetin, and kaempferol against the core target, mTOR, which was identified by amino acid metabolic screening. This evaluation was aimed at assessing the affinity of these active ingredients to their targets. The docking results showed that the binding energies of trehalose, morroniside, loganin, hypaconitine, quercetin, and kaempferol to the core target are all below −5.0 kJ/mol. This suggests a strong binding capability of these compounds to mTOR. Notably, hypaconitine (−8.9 kJ/mol), quercetin (−8 kJ/mol), and kaempferol (−8.7 kJ/mol) exhibited the highest binding affinities toward mTOR (Figure 7).

## 4 Discussion

Infertility represents a significant global reproductive health concern, with male infertility accounting for approximately 60% of all infertility cases (15, 16). Asthenozoospermia is recognized as a major contributor to male infertility (17). Although there are many empirical treatments available, such as hormone therapy and antioxidant therapy, these tend to produce certain side effects (6, 17). The pathogenesis of asthenozoospermia is multifaceted. Given the complexity of traditional Chinese medicine (TCM) formulas, which comprise various compounds and targets, it may be beneficial to consider TCM for the treatment of idiopathic infertility to enhance semen quality and promote natural fertility (18). Yougui Pill has been used in several ancient TCM formulations for the treatment of osteoarthritis. It is a widely used preparation within TCM designed to addressing osteoporosis associated with kidney yang deficiency, consisting of Chinese medicine ingredients that tonify the kidney and nourish yin (19). Furthermore, Yougui Pill combined with Buzhong Yiqi Decoction have demonstrated efficacy in alleviating female sexual dysfunction by modulating intestinal flora in ovariectomized (OVX) rats (20). Additionally, Yougui Pill can be used as a therapeutic target for treating kidney yang deficiency by altering the gut microbiota (21). Among its components, Wolfberry is primarily indicated for addressing decreased sexual and reproductive performance; Dodder-wolfberry represents a core drug pair found in numerous classical prescriptions for treating male infertility (22). Various Chinese herbal ingredients such as dodder, *Epimedium*, and *Rehmannia glutinosa* have been shown to reduce reactive oxygen species levels and improve sperm motility. It should be noted that the cost of antler gum included in Yougui Pill is relatively high due to significant expenses associated with animal sourcing. Consequently, we selected MYP as our research subject.

Initially, UPLC-MS/MS was used to identify the active ingredients present in MYP. These compounds include trehalose, morroniside, maltotriose, shanzhiside methyl ester, oxoglutaric acid, hypaconitine, loganin, adenosine, gallic acid, quercetin, L-Arginine, catalpol, arbutin, kaempferol, L-Tryptophan, among other compounds. Studies have shown that trehalose is a non-reducing disaccharide derived from glucose (23), which exists within the extract of Chinese yam water (CYW) and exhibits protective effects against ethanol-induced gastric injury both *in vitro* and *in vivo*. It has been used to improve the quality of thawed mammalian sperm, exhibiting antioxidant properties that protect sperm from reactive oxygen species (ROS) damage. Additionally, it is one of the commonly employed cryoprotectants in sperm vitrification freezing (23). Furthermore, it has shown a protective effect against lipid peroxidation in calf testicular tissue (24). Morroniside belongs to the class of cyclic enol ether glycosides and serves as an active ingredient in the traditional Chinese medicine *Cornus officinalis* (25). This small molecule monomeric compound has been shown to possess multiple biological effects, including anti-inflammatory, anti-apoptotic, and antioxidative stress activities (26, 27). Loganin is a cyclic iridoid glycoside isolated from the fruit of *Cornus officinalis* and exhibits various cytoprotective functions (28). Research have shown that loganin prevents cell apoptosis by inhibiting ROS production and NLRP3 inflammasome activation while also demonstrating antioxidant effects (29). Moreover, it can attenuate liver and kidney damage as well as diabetes-related complications caused by metabolic abnormalities induced by oxidative stress, inflammation, and apoptosis (30). Loganin exerts beneficial anti-inflammatory effects through downregulation of the expression of TNF-*α*, MCP-1, and IL-6, while inhibiting the activation of the NF-κB signaling pathway (28). It has been found to ameliorate diabetes-induced reproductive damage by improving metabolic parameters associated with diabetes, protecting testicular structure and function, as well as reducing oxidative stress, inflammation, and apoptosis within the testes (31). The diester diterpenoid alkaloid represented by hypaconitine not only constitutes the main toxic component in Aconitum but also plays a significant role as an important pharmacodynamic component within this genus (32). Quercetin and kaempferol are prevalent in traditional Chinese medicinal herbs such as wolfberry, dodder, and raspberry. Their interventions for spermatogenic disorders mainly target oxidative stress management, ROS metabolism regulation, and modulation of the NF-κB signaling pathway (33, 34). As representatives of flavonoids, both quercetin and kaempferol demonstrate excellent antioxidant properties as well as anti-inflammatory effects; they possess anticancer activity alongside antibacterial and neuroprotective characteristics (35). Furthermore, they may contribute to preventing and treating spermatogenic disorders by attenuating oxidative stress and inflammation while maintaining cytoskeletal integrity (36). Previous studies have indicated that quercetin can indirectly influence sexual organ stimulation at the cellular and organ levels while positively affecting serum testosterone levels (37). Additionally, kaempferol treatment has been shown to normalize reproductive hormones and enhance sperm function parameters in poisoned rats (38). These findings suggest that MYP treatment for OA may be associated with these active components.

The proper functioning of spermatogenesis within the testes necessitates normal levels of sex hormones. Follicle-stimulating hormone (FSH) and luteinizing hormone (LH) secreted by the pituitary gland act upon interstitial cells as well as Sertoli cells in the testes; this interaction promotes increased secretion of testosterone (T) and estradiol (E2), which in turn promotes differentiation between spermatogonial cells. Serum sex hormone levels are indicative of testicular spermatogenesis, and in infertile men, these hormone levels are often disrupted (39). The study further confirmed the impact of MYP on oligoasthenozoospermia (OA) in Simmental bulls, revealing that MYP administration significantly enhanced semen quality as well as hormone levels such as estradiol (E2), testosterone (T), and luteinizing hormone (LH). Studies have demonstrated that serum concentrations of testosterone (T), follicle-stimulating hormone (FSH), and inhibin B (INHB) in patients with oligoasthenozoospermia are typically lower than normal ranges, while luteinizing hormone (LH) tends to be elevated (5). DRLC treatment significantly reversed the PCB-induced elevation of FSH, LH, and testosterone levels in a rat model (40), consistent with the results of previous studies.

Semen metabolomics is considered a promising approach to identify biomarkers associated with semen quality and fertilization ability, as well as to elucidate drug mechanisms of action (41). Major metabolic alterations associated with male infertility due to semen abnormalities include energy-related metabolic pathways such as glycine, serine, and threonine metabolism, as well as arginine and proline metabolism (42). Notably, amino acid deficiencies may adversely affect the energy metabolism of spermatozoa, thereby severely impairing sperm viability. The status of amino acid metabolism directly reflects the metabolism of sex hormones; specifically, amino acid metabolism is reduced in the semen of asthenozoospermia patients (43). A recent study identified 63 metabolites as potential biomarkers for male infertility; among these metabolites, several amino acid derivatives were associated with various semen parameters (44). Additionally, reduced concentrations of metabolites within seminal plasma, including amino acids like lactate, citrate, creatinine, alpha-ketoglutarate, spermine, and putrescine, have been used to differentiate between OA and normal sperm controls (45). Asthenozoospermia (AS) is associated with the metabolism of aspartate, the methionine cycle, the urea cycle, and branched-chain amino acid metabolism. Patients diagnosed with AS exhibit reduced levels of phenylalanine (Phe) and tyrosine (Tyr) in their blood (14). Therefore, in the present study, amino acid metabolomics of semen from Simmental bulls was analyzed and it was found that administration of MYP reversed various metabolic changes. Notably, t Notably, the expression levels of D-aspartate, D-cysteine, D-serine, D-tryptophan, D-tyrosine, D-arginine, L-citrulline, L-serine, L-tryptophan, and L-tyrosine were increased. The majority of differentially expressed genes were enriched in multiple amino acid metabolic pathways; this suggests a potential role for MYP in regulating amino acid metabolism. Further enrichment analysis of the differential metabolites indicated that MYP mainly influences cysteine and methionine metabolism as well as glycine, serine and threonine metabolism; alanine, aspartate, glutamate metabolism; arginine biosynthesis; general amino acid biosynthesis; D-amino acid metabolism; along with phenylalanine-, tyrosine-, and tryptophan-biosynthesis pathways. Amino acids can be regarded as significant biomarkers for oligospermia. Previous studies have demonstrated that amino acids play a crucial role in the synthesis of bioactive substances such as reproductive hormones and coenzymes involved in various energy metabolic processes (43). D-Aspartic acid is present in stromal, Sertoli, and germ cells, particularly spermatogonia, as well as elongated spermatocytes and spermatozoa from both rodent models and human testes. A strong correlation exists between levels of D-aspartic acid (D-Asp) and testosterone concentrations within rat testes (46). The observation that decreased testicular D-Asp levels were accompanied by decreased serum testosterone levels and testosterone biosynthesis-related enzymes is consistent with our findings (47). Additionally, D-ribose-L-cysteine (DRLC) exhibits potential reversal effects on PCR-induced testicular damage within 30 days (40). Adding arginine to the feed can enhance the number of spermatogonia, elevate serum nitric oxide (NO) levels, and increase the abundance of testicular arginine and putrescine. This supplementation subsequently improves testicular development and semen quality in boars (48). Studies have shown that compound amino acid capsules exhibit significant clinical efficacy in patients with asthenozoospermia. Following treatment with these capsules, there was an observed increase in the expression level of Nrf2 pathway proteins within patients’ sperm (49). Elevated levels of amino acids and lipids fulfill the high energy requirements during spermatogenesis, and arginine is essential for sperm production. Supplementation with arginine may contribute to improved healthy sperm counts and enhanced fertility (42). The results of the present study indicated that multiple amino acids exhibited an upward trend following MYP treatment, suggesting that MYP can ameliorate oligospermia (OA) in Simmental bulls by modulating amino acid metabolism.

The analysis of the regulatory network governing amino acid metabolism revealed involvement of the mTOR signaling pathway. Previous research has established that this pathway plays a key role the signaling network by balancing metabolic signals related to growth factors, energy status, oxygen availability, stress response, and amino acids. It is capable of initiating appropriate cascade events based on various stimuli leading to either protein and lipid synthesis or autophagy (50). High doses of follicle-stimulating hormone (FSH) activate autophagy through inhibition of the AKT–mTOR signaling pathway; this process enhances estradiol (E2) production while increasing FSH receptor (FSHR) expression and reducing bovine ovarian granulosa cell activity (51). Inhibition of mTOR signaling triggers the activation of autophagy in rat testes as a self-protective mechanism against external stress (52). It is hypothesized that sustained activation of mTOR may be implicated in OA pathogenesis; therefore, MYP may alleviate OA by influencing associated cytokines and regulating mTOR signaling pathways.

## 5 Limitations

The primary limitation of this study is the small sample size of Simmental bulls, which may affect the generalizability of the results, primarily due to the high cost and limited availability of genetically pure Simmental bulls under controlled feeding conditions. However, these bulls were rigorously selected as representative individuals with homogeneous genetic backgrounds and standardized management conditions, reducing within-group variability. Future studies with larger cohorts from multiple farms are needed to validate our findings.

## 6 Conclusion

The major compounds present in MYP include trehalose, morroniside, maltotriose, shanzhiside methyl ester, oxalic acid and other compounds. MYP treatment has been shown to enhance sperm motility, increase sperm density, and elevate levels of testosterone (T), estradiol (E2), and follicle-stimulating hormone (FSH) in Simmental bulls with osteoarthritis (OA). The results of amino acid metabolomics revealed a total of 47 upregulated amino acids and 20 downregulated amino acids, indicating that MYP can restore the balance of amino acid metabolism. Furthermore, MYP appears to regulate multiple metabolic pathways, including cysteine and methionine metabolism, glycine, serine, and threonine metabolism, alanine, aspartate, and glutamate metabolism, arginine biosynthesis, as well as the mTOR signaling pathway. Molecular docking validated robust binding interactions between the main active ingredients and respective core targets. These findings suggest that MYP may improve OA by modulating amino acid metabolism.

## Data Availability

The raw data supporting the conclusions of this article will be made available by the authors, without undue reservation.

## Footnotes

^1^ https://old.tcmsp-e.com/tcmsp.php

^2^ https://www.rcsb.org/
