# Supplementary figures and images for: Effect and mechanism of modified Yougui power on Simmental bulls with oligoasthenozoospermia based on targeted amino acid metabolism
Source: Front Vet Sci. 2025 Jun 18;12:1595145. doi: 10.3389/fvets.2025.1595145 (PMC12217934; doi:10.3389/fvets.2025.1595145)

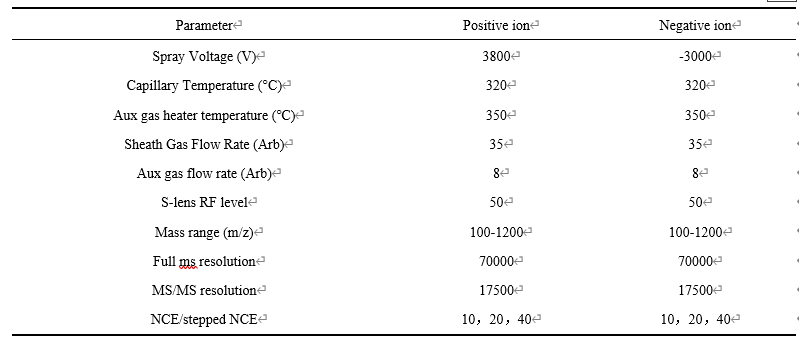

Supplement: Supplementary file 1 [file Image_1.TIFF]
